# Identification, Expression Patterns and RNA Interference of Aquaporins in *Dendroctonus armandi* (Coleoptera: Scolytinae) Larvae During Overwintering

**DOI:** 10.3389/fphys.2019.00967

**Published:** 2019-08-02

**Authors:** Danyang Fu, Lulu Dai, Haiming Gao, Yaya Sun, Bin Liu, Hui Chen

**Affiliations:** ^1^College of Forestry, Northwest A&F University, Yangling, China; ^2^State Key Laboratory for Conservation and Utilization of Subtropical Agro-Bioresources, Guangdong Key Laboratory for Innovative Development and Utilization of Forest Plant Germplasm, College of Forestry and Landscape Architecture, South China Agricultural University, Guangzhou, China

**Keywords:** aquaporins, *Dendroctonus armandi*, cold tolerance, qRT-PCR, expression, RNAi, mortality

## Abstract

The ability to survive annual temperature minima could be a key determinant of distribution limits for insects under global climate change. Recent studies have suggested that insect aquaporins are indispensable for cellular water management under conditions that lead to dehydration and cold stress. Aquaporins are integral membrane water channel proteins in the major intrinsic protein superfamily and promote selected solutes and the movement of water across biological membranes. We cloned and characterized nine full-length aquaporins from *Dendroctonus armandi* (DaAqps), the most destructive forest pest in the Qinling Mountains of Shaanxi Province, China. Eight of the DaAqps belong to three classical aquaporin grades, including the *Drosophila* integral protein, the *Pyrocoelia rufa* integral protein, the entomoglyceroporins and one that belongs to the unorthodox grade of aquaporin 12-like channels. The DaAqps were increasingly expressed during different developmental stages and in different larval tissues, and expression peaked in mid-winter. They were tested under cold conditions for different lengths of time, and the expression of almost all DaAqps was down regulated with decreasing temperatures and long-term exposure to cold conditions. However, when the lowest temperatures were reached, the levels were immediately upregulated. These genes indicate that cold tolerance can improve through mortality responses at low temperatures after RNA interference of DaAqps. In our study, we analyzed the molecular response, expression patterns, and RNA interference of DaAqps and clarified the crucial role of protective compounds (aquaporins) underlying *D. armandi* cold tolerance and provide a new pest control method.

## Introduction

Bark beetles (Curculionidae: Scolytinae), especially *Dendroctonus* species have been shown to cause heavy damage to natural and coniferous forests. The Chinese white pine beetle *Dendroctonus armandi* Tsai and Li destroys *Pinus armandi* Franch and is considered to be the most destructive forest pest in the Qinling and Bashan Mountains of Shaanxi Province, China (Dai et al., 2014). *D. armandi* directly attacks healthy *P. armandi* trees over 30 years of age but has also been shown to indirectly damage host trees, attracting other pests to the host trees, causing even greater ecological and economic (Chen and Tang, 2007). The voltinism of *D. armandi* in the Qinling and Bashan Mountains is altitude dependent (Wang et al., 2010). Commonly, there are two generations per year at altitudes lower than 1,700 m, three within 2 years from 1,700 to 2,150 m, and only one per year above 2,150 m (Zhao et al., 2017). Furthermore, ambient temperature decreases with increasing altitude. Therefore, extremely low temperatures coupled with high altitudes challenge the survival of insects (Lee, 1989; Koštál and Šimek, 2000). In recent years, low winter temperatures have limited insect population size, activity, and development with global climate change (Rosenberger et al., 2017). The ability to survive annual low temperatures could be a key determinant of insect distribution (Ungerer et al., 1999). Winter climate can also exert a strong selection pressure on physiological and behavioral attributes that promote overwinter survival (Kukal et al., 1991; Lombardero et al., 2000).

Thus, insects exposed to cold during low winter temperatures have developed diverse behavioral, physiological, and morphological adaptations (Bale, 2002; Clark and Worland, 2008). These adaptations protect cells from rapid fluctuations in weather (Kultz, 2003), including the production of protective compounds, such as heat shock proteins (Kelty and Lee, 2001; Rinehart et al., 2007; Li and Denlinger, 2008), aquaporins (AQPs) (Nielsen et al., 2005; Philip et al., 2008; Yi et al., 2011), and polyols (Koštál et al., 2007). The results of these previous studies suggest that AQPs might contribute to the cold tolerance of *D. armandi* because free water would be transformed into bound water to avoid the extracellular fluid freezing (Wang et al., 2016a).

Water channel proteins known as AQPs belong to the major intrinsic protein (MIP) superfamily. Several previous studies have demonstrated that AQPs are transmembrane proteins that promote selected solutes and movement of water across biological membranes (Tsujimoto et al., 2017). AQPs have been divided into two types, one of which is highly selective for the passage of water; and the other is involved in the transport of small neutral solutes (such as glycerol) as well as water, and it is inhibited by mercuric chloride (Preston et al., 1992; Maurel, 1997; Le Cahérec et al., 1996a,b). AQPs are found in all higher animals and plants, as well as some insects (e.g., the leafhopper *Cicadella viridis* and the mosquito *Aedes aegypti*) (Beuron et al., 1995; Pietrantonio et al., 2000). Recent studies suggest that insect AQPs are indispensable for cellular water management under conditions that lead to dehydration and cold stress (Philip et al., 2008; Xia et al., 2017). However, the physiological and molecular mechanisms of AQPs underlying tolerance to low temperatures in bark beetles remains poorly understood.

In the present study, to better understand the biological significance of AQPs response to cold stress in bark beetles, we isolated five AQPs from *D. armandi* and identified them in terms of phylogenetics, bioinformatics predictions, and expression patterns. The RNAi analysis also determined that AQPs play an important role in the response of *D. armandi* overwintering larvae to cold conditions.

## Materials and Methods

### Insect Sampling, Preparation, and Treatments

The larvae of *D. armandi* were collected from the Huangguan Forest Farm, Ningxi Forestry Bureau in Shaanxi, China. The sampling site was on the southern slope of the middle Qinling Mountains (33° 40′ N, 108° 34′ E) where the dominant conifers are the infested *P. armandi* trees, and we collected the larvae according to the five-point sampling method (Dai et al., 2014). The overwintering *D. armandi* larvae used in the experiments were collected on the 25th of each month between September 2017 and May 2018. Then they were transported to the laboratory in disposable petri dishes with moist paper and immediately frozen in liquid nitrogen and stored at –80°C until analysis. The larvae used for RNAi and the experiments under low temperature conditions were stored in the dark.

Seven life stages of *D. armandi* used in the experiment were collected: egg, larvae, mature larvae that had ceased feeding, teneral adults with a light body color, emerged adults, and feeding adults that were invading a new host. Adults were sexed by the seventh abdominal tergite and external genitalia (Lyon, 1958; Dai et al., 2014; Zhao et al., 2017; Li et al., 2018). We also dissected the heads, foreguts, midguts, hindguts, and body fat of overwintering larvae collected in February 2018 for analysis of expression in tissues.

For testing the DaAqps expression and the larvae mortality at different low temperatures, the larvae collected in January 2018 were put in a dry electro thermostat (Hangzhou Long Gene Scientific Instruments Co., Ltd., Zhejiang, China). Individual larvae were placed in 200 μl perforated Eppendorf PCR tubes and exposed to seven different temperatures (4, 0, –2, –4, –6, –8, and –10°C). The difference is that the exposure time points under different low temperatures in the DaAqps expression experiment are five (1, 6, 12, 18, and 24 h) and are seven in the mortality test (0.5, 1, 2, 3, 4, 5, and 6 h). After testing, the samples were kept at 25°C for 1 h until their body moved when they were stimulated with tweezers (Atapour and Moharramipour, 2009). We recorded the mortality at the end of the test, and the larvae that survived in the expression experiment were used for RT-qPCR. Twenty larvae were used for each treatment, including three replicates.

### Total RNA Isolation and cDNA Synthesis

Total RNA was isolated from insects using an UNlQ-10 Column Trizol Total RNA Isolation Kit (Sangon Biotech, Shanghai, China) according to the manufacturer’s protocol. The integrity of RNA was verified with 1.0% agarose gels electrophoresis and quantification by spectrophotometry in a NanoDrop 2000 (Thermo Fisher Scientific, Inc., Pittsburgh, PA, United States). The purity was considered as the mean of the A260/A280 μg/ml (A260 × dilution factor × 40). First-strand cDNA synthesis with carried out with the Fast King RT reagent Kit with gDNA Eraser (Tiangen, China), following the manufacturer’s instructions. Single-stranded 3′ and 5′ RACE cDNA was synthesized from mixed RNA (RNA at different developmental stages, 1 μg) using a SMARTer^®^ RACE 5′/3′ Kit (TaKaRa, Dalian, China) per specification, then stored at –20°C until analysis.

### DaAqps Amplification and Cloning

The synthesized cDNA was used as a template for the PCR. Specific primers (Supplementary Table S1) were designed by Primer Premier 5.0, based on transcriptome dataAQP sequences of other related species from GenBank. The cDNA amplification was carried out in a 20 μl reaction mixture containing 1 μl cDNA (1:4 dilution), 0.25 μm of each primer, 2 × EcoTaq PCR SuperMix (TransGen, Beijing, China), and sterile water. Then the PCR was carried out in a C1000 thermocycler (Bio-Rad, CA, United States) with the following cycling parameters: initial denaturation for 3 min at 94°C, followed by 30 cycles of 30 s at 94°C, 30 s at 45–60°C, 1 min at 72°C, and a final extension for 10 min at 72°C. PCR products were checked using a 1 × 4S Red Plus Nucleic Acid Stain (Sangong, Shanghai, China) in 1% agarose gels and a 2K plus DNA marker (TransGen, Beijing, China) for comparison. PCR amplified fragments were purified using a Gel Purification Kit (Omega Bio-Tek, Norcross, GA, United States), and connected with pMDTM 18-T vector (TaKaRa, Dalian, China), then transformed into DH5α chemically competent *Escherichia coli* cells (TransGen, Beijing, China). Transformants (white colonies) were selected on Amp/LB/Xgal/IPTG plates, using vector-specific primers (M13-F and M13-R) for positive clones from the PCR. Finally, high concentration bacterial suspensions of positive clones were sequenced by Augct Biotech, Beijing, China. Two independent clones were submitted to minimize potential PCR mutations. The obtained partial sequences were manually edited with the DNAMAN software to obtain inserts, which were then blasted against the NCBI database.

### RACE

#### 5′, 3′ RACE and Cloning of Full-Length cDNA

Gene-specific primers for 5′ and 3′ RACE (Supplementary Table S1) were designed based on the obtained sequence fragments. Touchdown PCR was used to improve the amplification specificity of the 5′-UTR and 3′-UTR sequences (annealing temperatures: 60–50°C). The amplified products were then subjected to repeat steps as described in the section “DaAqps amplification and cloning”. The full-length sequence was determined by assembling the cDNA fragments and the sequence obtained from the 5′ and 3′-RACE PCR. To obtain the full-length gene, we designed gene-specific primers encompassing the putative start and stop codons (Supplementary Table S1).

#### Full-Length cDNA Sequence Analysis and Comparison

Putative full-length gene sequences were deposited in the GenBank, and accession numbers are listed in Table 1. The open reading frames (ORFs) of cDNA gene sequences were obtained from ORF Finder^1^.

**TABLE 1:** Blast matches of putative DaAqps.

**Gene name**	**Blast matches in gene bank**			**Identify%**
	**Species**	**Gene**	**Accession no.**	
DaDripv1	*Dendroctonus ponderosae*	AQP × 1	XP_019764073.1	96
DaDrip_v2	*Dendroctonus ponderosae*	AQP × 2	XP_019764074.1	96
DaDrip_v3	*Dendroctonus ponderosae*	AQP × 3	XP_019764075.1	96
DaPrip	*Dendroctonus ponderosae*	AQP × 1	XP_019764042.1	94
DaEglpA1_v1	*Dendroctonus ponderosae*	AQP × 2	XP_019764160.1	90
DaEglpA1_v2	*Dendroctonus ponderosae*	AQP × 4	XP_019764163.1	92
DaEglpA1_v3	*Dendroctonus ponderosae*	AQP × 5	XP_019764165.1	93
DaEglpA2	*Dendroctonus ponderosae*	AQP	XP_019759732.1	97
DaAqp12L	*Anoplophora glabripennis*	AQP11	XP_018562473.1	57

Molecular mass (kDa) and isoelectric points were computed using the ProtParam tool^2^ (Li et al., 2018). Signal peptide analysis was performed using SignalP 4.1^3^. Glycosylation predictions were based on Biological Sequence Analysis^4^. Homologs of *D. armandi* (DaAqps) were identified with BlastP in the NCBI database^5^. Multiple sequence alignment with secondary structure element assignment of protein structure was exploited in ESPript^6^. TMHMM v. 2.0^7^ was used to intuitively show topological and transmembrane domain predictions. The phosphorylation sites were predicted by NetPhos 2.0^8^. Sequence alignment and phylogenetic analysis of DaAqps homologs from many insect species were carried out in MEGA 5.0 (Tamura et al., 2011).

### RT-qPCR

The Roche SYBR green system (Roche Diagnostics GmbH, Mannheim, Germany) and the CFX-96 real-time PCR Detection System (Bio-Rad, CA, United States) were employed for RT-qPCR. We found that CYP4G55 (accession number: JQ855658.1) and β-actin (accession number: KJ507199.1) were more stable than 18S rRNA. Therefore, the expression level of AQPs was normalized by geometric averaging of these two control genes (Vandesompele et al., 2002). Specific RT-qPCR primers were designed in Primer Premier 5.0 based on obtained nucleotide sequences (Supplementary Table S1). The amplification efficiency and validity of each gene, relative standard curves were plotted between the mean values of quantification cycles at different dilutions (1.0, 10-1, 10-2, 10-3, and 10-4) of cDNA, and the efficiency of the primers was estimated to be 100 ± 5%. R2 values were used to directly evaluate the PCR validation and were >0.95. Furthermore, a melting curve reaction was used to estimate their specificity. The amplification efficiency of primers was computed using the PCR reactions, which were carried out in a 20 μl mixture containing 10 μl of 2 × SYBR Premix Ex Taq (Roche Diagnostics GmbH, Mannheim, Germany), 3 μl of cDNA (diluted four times), 0.8 μl of each primer, and 5.4 μl of nuclease-free water. The three-step thermocycling conditions used were as follows: 95°C for 10 min, followed by 40 cycles of 95°C for 10 s, annealing temperature of DaAqps each pair of primers for 10 s, 72°C for 20 s; melting curve analysis at 95°C for 10 s, 65°C to 95°C in increments of 0.55°C for 5 s. Three biological and three technical replicates were included to ensure reproducibility. Relative expression of DaAqps was determined using the 2^–ΔΔCt^ method (Livak and Schmittgen, 2001; Yuan et al., 2007; Dai et al., 2014).

### RNAi

#### The dsRNA Synthesis and Injection

The T7 Ribo-MAX^TM^ Express RNAi System (Promega, Madison, MI, United States) was used for the composition of dsRNA. RNAi primers (Supplementary Table S1) were designed based on the full-length DaAqps. The final dsRNA products (diluted to 1000 ng/μl in diethyl pyrocarbonate (DEPC)-treated water) were stored at –80°C and used within 6 months.

Before injection, *D*. *armandi* larvae were placed in an ice bath for 10 min. The larvae were immobilized on an agarose plate using manual forceps (Wang et al., 2016b). Afterward, each *D. armandi* larvae was injected with 0.05 μl DEPC treated water or dsRNA solution (200 ng/μl), using Hamilton Microliter^TM^ syringes with 32 G sharp-point needles (Hamilton, Bonaduz, Switzerland) (Chen et al., 2010; Tian et al., 2012; Choi et al., 2014). The untreated larvae were used as the control group for the experiment. Each treatment group contained 80 larvae. Following injections, the larvae were kept at 4°C in a refrigerator. Nine larvae were removed at different time intervals (24, 48, and 72 h) from each treatment group and frozen in liquid nitrogen for storage at –80°C until RT-qPCR analysis.

#### Low-Temperature Resistance Test

Larvae were injected after 72 h, and then left at room temperature for 1 h. The larvae that did not move were considered to be dead (Ma et al., 2006). Larval survival and mortality were tested under the treatments and control conditions for 1 h to determine the responses to low temperature (4, 0, –2, –4, –6, –8, and –10°C). The test included three biological replicates.

### Statistical Analysis

Data from RT-qPCR and mortality at low temperature were analyzed with SPSS 19.0 (IBM, Chicago, IL, United States). Significant differences between treatments in DaAqps mRNA levels and mortality were calculated using an ANOVA (*p* < 0.05), and then the results were adjusted using a Duncan’s multiple-comparison test. Graphs were plotted in Prism 6.0 (GraphPad Software, CA, United States) and OriginPro 2017 (Origin ECN, Melbourne, VIC, Australia).

## Results

### DaAqp Sequences and Bioinformatics Analysis

#### Identification of Aquaporin Genes

In total, we identified nine sequences that were corresponded to the AQPs from five *D. armandi* transcriptomes. The full-length AQP gene sequences shared the highest identity (90–97%) with the bark beetle *D. ponderosae*, except for DaAqp12L (Table 1), which shared 57% identity with AQP11 from the Asian long-horned beetle *Anoplophora glabripennis*.

#### Sequence Characteristics

We identified three N-terminal isoforms of *Drosophila* integral protein (Drip) for *D. armandi* (DaDrip) (variants 1, 2, and 3) by 5′ RACE PCR. Each DaDrip CDS encoded 254, 249, and 245 amino acids, respectively (Table 2). *Pyrocoelia rufa* integral protein (Prip) for *D. armandi* (DaPrip) consisted of an 828-bp CDS that encoded 275 amino acids, with molecular weights (MW) and isoelectric points (IP) of 28.967 kDa and 7.75, respectively. *D. armandi* also contained the Eglps subfamily of clade A (DaEglpA) (Ekert et al., 2016), and we identified two unique DaEglpAs by gene cloning. DaEglpA1 contained the three isoforms, variants 1, 2, and 3, which encoded 954, 783 and 762 amino acids, respectively. MW and IP of DaEglpA2 were 26.438 kDa and 8.26, respectively (Table 2). DaAqp12L is in a different subfamily from *D. armandi* with 870-bp CDS and MW of 31.765 kDa.

**TABLE 2:** Physicochemical properties of DaAqp sequences.

**Gene name**	**Accession no**.	**Full-length (bp)**	**ORF^a^ size (aa/bp)**	**MW^a^ (KDa)**	**IP^a^**
DaDrip_v1	MH579720	1113	254/765	27.054	6.03
DaDrip_v2	MH579721	1280	249/750	26.438	6.03
DaDrip_v3	MH579722	1185	245/738	25.994	6.03
DaPrip	MH579719	1220	275/828	28.967	7.75
DaEglpA1_v1	MH579723	1341	317/954	34.904	7.10
DaEglpA1_v2	MH579724	1127	260/783	28.323	5.88
DaEglpA1_v3	MH579725	1087	253/762	27.417	6.04
DaEglpA2	MH579726	1337	271/816	26.438	8.26
DaAqp12L	MH579727	948	289/870	31.765	8.22

*^*a*^ORF, open reading frame; IP, isoelectric point; MW, molecular weight, predicted by ORF Finder and Signal P 4.1 Server.*

**FIGURE 1:**
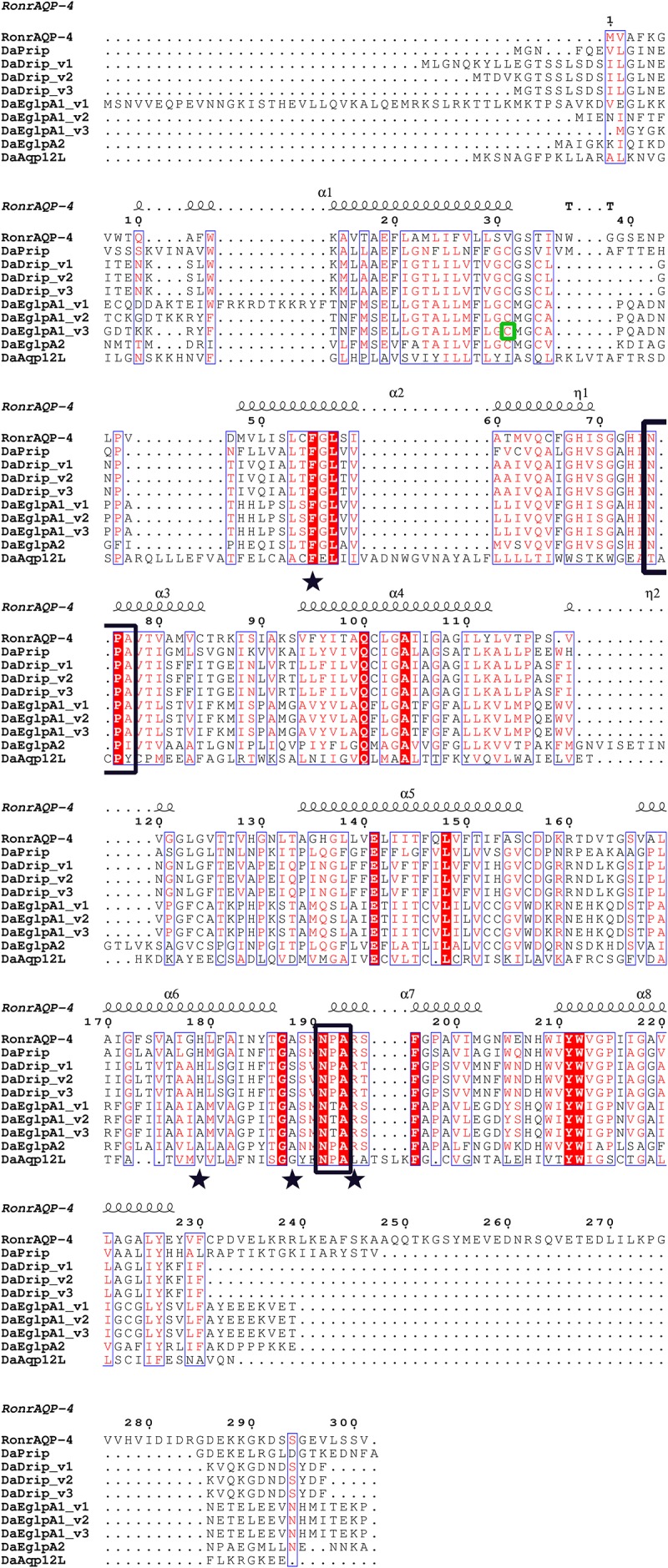
Multiple sequence alignment and secondary structure element assignment. The alignment included AQP genes from *Dendroctonus armandi* and *Rattus norvegicus* AQP4. NPA motifs are shown with black boxes and residues that correspond to the ar/R constriction site are shown as stars. A predicted mercury sensitive cysteine (Cys31) from DaEglpA1_v3 is shown with green box.

DaDrip and DaPrip had two Asn-Pro-Ala (NPA) motifs like the other members of the AQP superfamily MIP (Murata et al., 2000). While DaEglpA1 and DaEglpA2 had one NPA motif, the other motif comprised Asn-Thr-Ala and Asn-Pro-Ile, respectively (Figure 1). In particular, DaAqp12L presented two NPA motifs similar to other insect AQPs, but they were different in other subfamilies (Campbell et al., 2008). An NPA motif of DaAqp12L was Cys-Pro-Tyr, and the other was like that of the AQPs (Figure 1). The four residues comprised the ar/R selectivity filter (Phe, His, Ala, Ser, Asn, or Arg) which is conserved in all DaAqp-translated sequences, except for that of DaAqp12L (Figure 1). Especially for DaEglpA1_v3, which has an *N*-palmitoyl cysteine in position 31 (Figure 1). All DaAqps have *O*-glycosylation in different positions but do not have *C*-glycosylation (Table 3). In addition, we predicted 6–7 transmembrane topology domains in total and many phosphorylation sites in all DaAqp genes (Table 3).

**TABLE 3:** Prediction of transmembrane topology, phosphorylation sites and glycosylation for DaAqp sequences.

**Protein name**	**The predicted transmembrane helices^a^**			**Predicted phosphorylation sites**				**Glycosylation predictions**	
	**Number**	**Consensus position**		**The number of residues**			**Total**	***N*^b^**	***O*^c^**
		**N-terminus**	**C-terminus**	**Serine**	**Threonine**	**Tyrosine**			
DaDrip_v1	6	Inside	Inside	10	5	2	17	HINPAVTIS	Ser201,Ser211
DaDrip_v2	6	Inside	Inside	11	6	2	19	HINPAVTIS	Ser206,Ser196
DaDrip_v3	6	Inside	Inside	11	5	2	18	HINPAVTIS	Ser202,Ser192
DaPrip	6	Inside	Inside	4	5	1	10	HLNPAVTIG	Ser199
DaEglpA1_v1	6	Inside	Inside	8	12	1	21	HLNPAVTLS	Ser45
DaEglpA1_v2	6	Inside	Inside	6	11	0	17	HLNPAVTLS	–^d^
DaEglpA1_v3	6	Inside	Inside	6	9	0	15	HLNPAVTLS	−
DaEglpA2	6	Inside	Inside	7	3	0	10	–	Thr151
DaAqp12L	7	Outside	Inside	11	10	3	24	–	Ser62

*^*a*^The amino and carboxylterminal ends of DaAqp sequences were predicted to be either intracellular or extracellular based on consensus positions. ^*b*^*N*-glycosylation. ^*c*^*O*-glycosylation. ^*d*^No result.*

#### Phylogenetic Analysis

**FIGURE 2:**
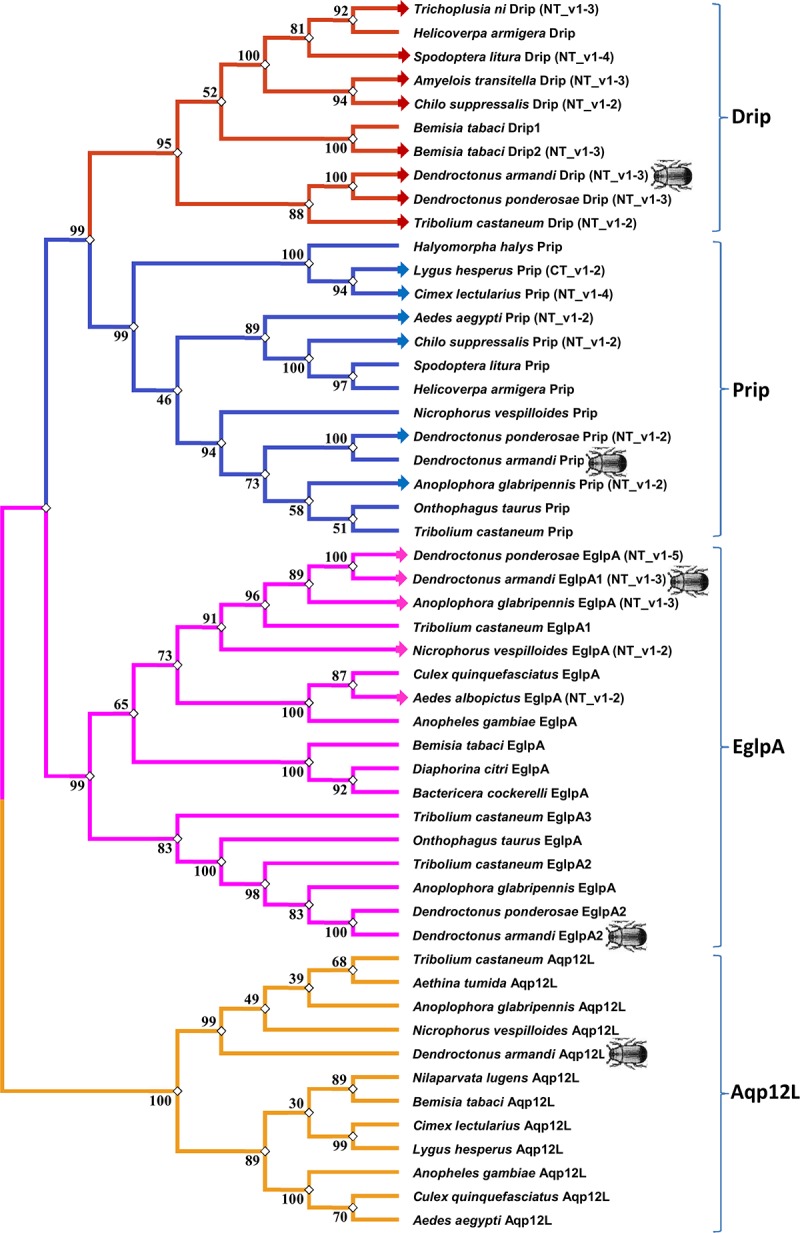
Phylogenetic analysis and classification of DaAqps. The phylogenetic tree analysis of DaAqps used the Maximum Likelihood method by using the amino acidic substitution model WAG++G+I+F122 in MEGA5.0. The bootstrap consensus tree inferred from 1000 replicates. The analysis concerned 52 amino acid sequences and DaAqps were marked with insect images. Representatives from four major arthropod subfamilies (Drips, Prips, Eglps, and Aqp12L) are shown.

The maximum likelihood method was used in MEGA 5.0 for the phylogenetic analysis of DaAqps and other arthropod AQPs (Figure 2). Recently, invertebrate AQPs were grouped into six major subfamilies: the big brain cation channel proteins, Drip, Prip, Eglps, the distantly related Aqp12L, and Glps (Kambara et al., 2009; Goto et al., 2011; Stavang et al., 2015; Ekert et al., 2016). Phylogenetic analysis of full-length DaAqps with 43 other invertebrate AQPs from GenBank showed that DaAqp genes were separated into four distinct subfamilies: Drip, Prip, EglpA, and Aqp12L (Figure 2). DaAqps not only have single copies of the Drip, Prip, and Aqp12L subfamilies, but also have duplicated members of EglpA (DaEglpA1 and DaEglpA2). For DaAqps, there were three isoforms of Drip (DaDrip_v1, DaDrip_v2, and DaDrip_v3), and of EglpA1 (DaEglpA1_v1, DaEglpA1_v2, and DaEglpA1_v3), which have the alternatively spliced N-terminal domain.

### Expression Patterns of DaAqps

#### Expression of DaAqps in Different Developmental Stages and Tissues

**FIGURE 3:**
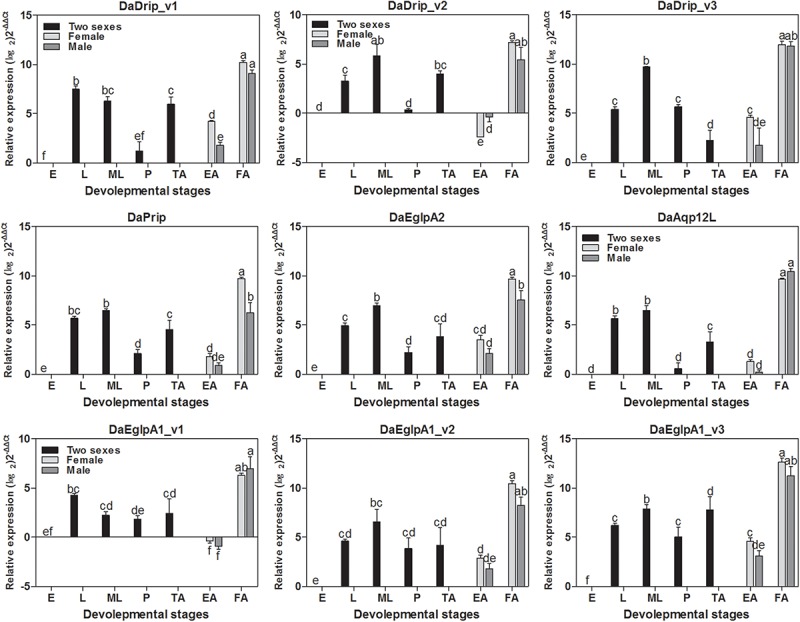
Relative mRNA expression levels of DaAqps in different developmental stages. The relative expression levels were normalized with actin and *CYP4G55* using the expression levels in the egg for calibration. The standard errors of the means of three biological replicates are represented by error bars. Significant differences between development stages DaAqps are marked with letters (*p* < 0.05, one-way ANOVA). All values are mean ± *SE*, *n* = 3. E, egg; L, larvae; ML, mature larvae; P, pupae; TA, teneral adult; EA, emerged adult; FA, feeding adult.

DaAqps were expressed in all developmental stages of *D. armandi* and were highly expressed in feeding adults and larvae (both young and mature larvae) but were less abundant in the other stages. Furthermore, we found that DaAqps expression in females was evidently higher than that in males, except for DaEglpA1_v1 and DaAqp12L (Figure 3).

**FIGURE 4:**
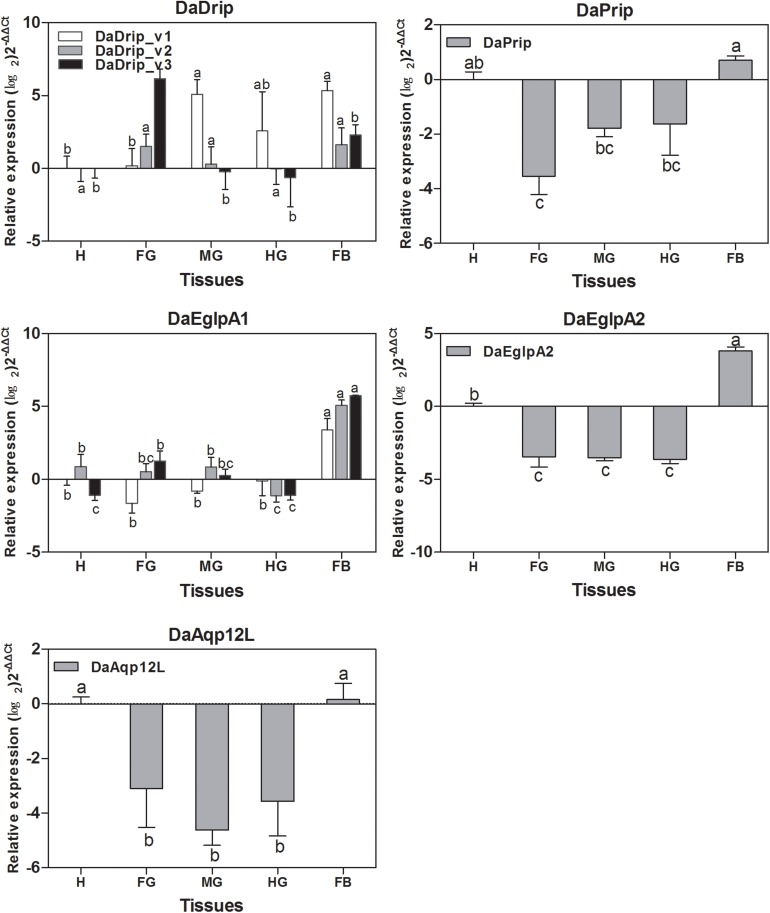
Relative expression levels of DaAqps in different tissues from larvae collected in February 2018. The relative expression levels were normalized with actin and *CYP4G55* using the expression levels in the head for calibration. The standard errors of the means of three biological replicates are represented by error bars. Significant differences between tissue DaAqps are marked with letters (*p* < 0.05, one-way ANOVA). All values are mean ± SE, *n* = 3. H, head; FG, foregut; MG, midgut; HG, hindgut; FB, fat body.

All DaAqps were expressed at different levels across multiple tissues. Except for DaDrip_v3, all other AQPs were highly expressed in the fat body, and DaEglpA1_v3 expression was predominant in this tissue. The highest expression of DaDrip_v3 was observed in the foregut. In the hindgut, only DaDrip_v1 was significantly expressed. All DaDrip and DaEglpA were not abundant in the head. In the midgut, DaDrip_v1 was highly expressed, while DaEglpA2 and DaAqp12L were only expressed at low levels. DaPrip, DaEglpA2, and DaAqp12L, all exhibited much lower expression in the digestive tract including the foregut, midgut, and hindgut (Figure 4).

#### Expression of DaAqps in Different Seasons

**FIGURE 5:**
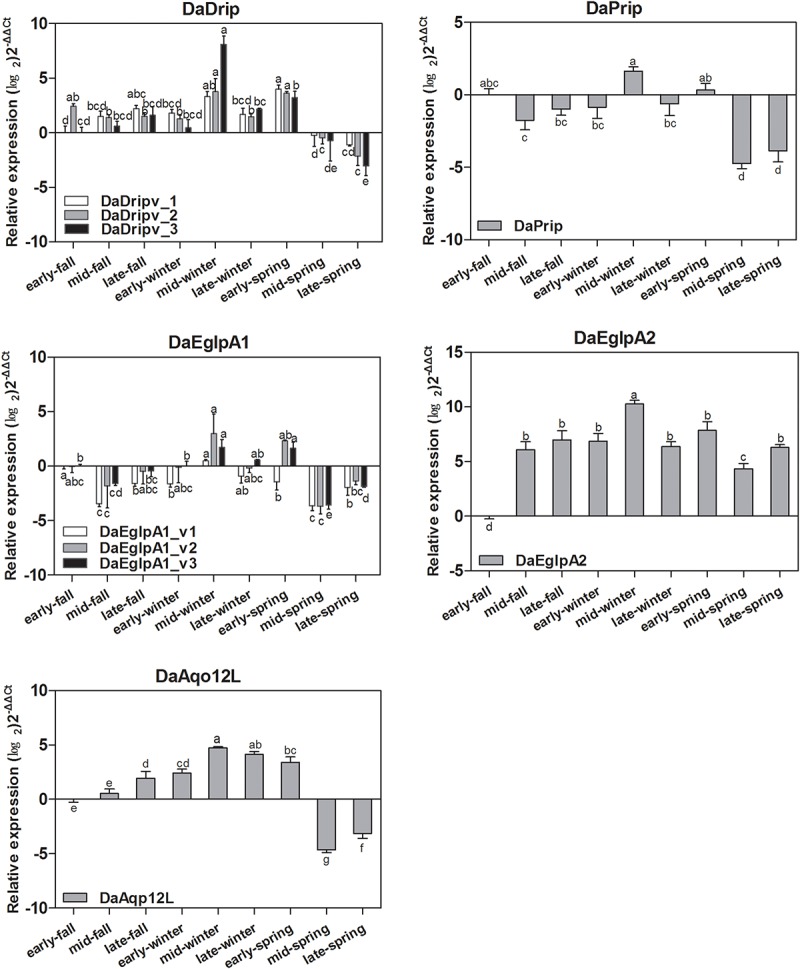
Seasonal relative expression levels of DaAqps. The relative expression levels were normalized with actin and *CYP4G55* using the expression levels in September for calibration. The standard errors of the means of three biological replicates are represented by error bars. Significant differences in each month DaAqps are marked with letters (*p* < 0.05, one-way ANOVA). All values are mean ± SE, *n* = 3.

Real-time PCR analysis was used to detect the transcript levels of DaAqps in different seasons from September 2017 to May 2018 (Figure 5). The level of DaAqps was upregulated in from fall to mid-winter (October to January). Significant downregulation of DaAqps was observed in spring. Apart from DaEglpA1_v1 and DaAqp12L, almost all DaAqps exhibited peak expression in mid-winter after a low expression in early winter. All other AQPs showed a lower expression in late-winter than in early spring except DaAqp12L (Figure 5). These results indicated that DaAqps were highly expressed in overwintering insects.

#### Expression Analysis of DaAqps Under Low Temperatures

**FIGURE 6:**
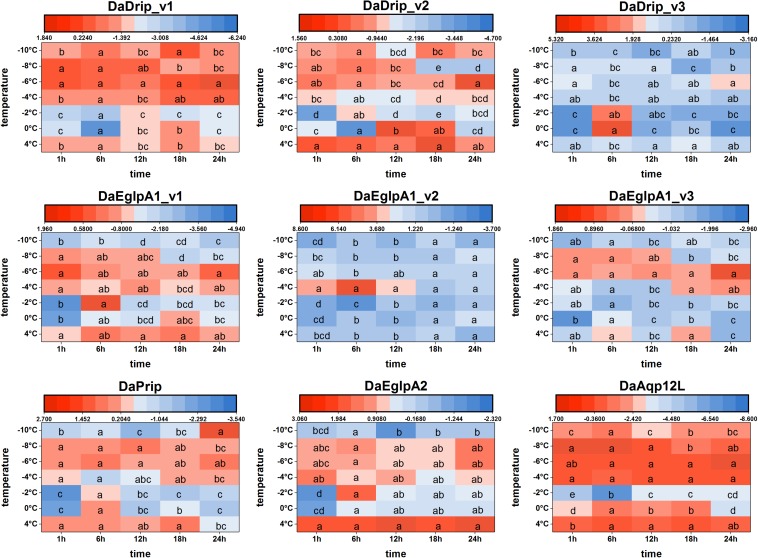
Relative mRNA expression levels of DaAqps under low temperatures. The relative expression levels were normalized with actin and *CYP4G55* using the expression levels in the January for calibration. Significant differences between changes of expression are marked with letters (*p* < 0.05, one-way ANOVA). All values were mean ± SE, *n* = 3.

We found that DaAqps expression diversified when larvae were exposed to cold conditions (Figure 6).

At 4°C, DaDrips expression was upregulated at 1, 6, 12, 18, and 24 h. Conversely, DaDrip expression was downregulated at –2°C. Also, DaDrip_v1 expression was highly up-regulated from –4 to –10°C, and DaDrip_v2 expression was highly up-regulated from –6 to –10°C. DaDrip_v3 was not highly expressed at most temperatures, however, it was highly expressed when larvae had exposed to 0°C for 6 h. In other words, DaDrips expression increased with decreasing temperature until being exposed to –10°C for 24 h (Figure 6).

The expression of DaPrip exhibited a similar trend to that of DaDrips at 4°C, and it was almost the same at different time points. Initially, expression decreased at 0 and –2°C, and then increased from –4 to –8°C. Finally, the level of DaPrip expression decreased again at –10°C (Figure 6).

DaEglpA expression responded in different ways when exposed to cold temperatures for different periods of time. From 0 to –8°C, DaEglpA1_v1 and DaEglpA2 expression initially decreased and then increased with decreasing temperature, and their expression continuously decreased at –10°C. Conversely, the expression of DaEglpA1_v3 decreased from 4°C to –4°C, and then was highly up-regulated at –6 and –4°C. DaEglpA1_v2 was not highly expressed at most temperatures, however, it was highly expressed when the larvae were at –4°C between 1 and 6 h. Consistently, the expression of the three DaEglpA1s was all downregulated at –10°C (Figure 6).

DaAqp12L expression exhibited a similar trend to that observed in DaDrip_v1. The high expression of DaAqp12L was observed at 4°C at all time points, and the lowest expression was observed at –2°C after 6 h. From –4 to –10°C, the level of DaAqp12L expression gradually decreased but was more highly expressed than it was at –2°C (Figure 6).

In general, among the different times tested under cold conditions, the expression of DaAqps decreased at lower temperatures until a critical temperature was reached, at which point they increased before decreasing again. In addition, the expression of DaEglpA1s expressions was lower throughout the experiment.

### Mortality at Low Temperature

**FIGURE 7:**
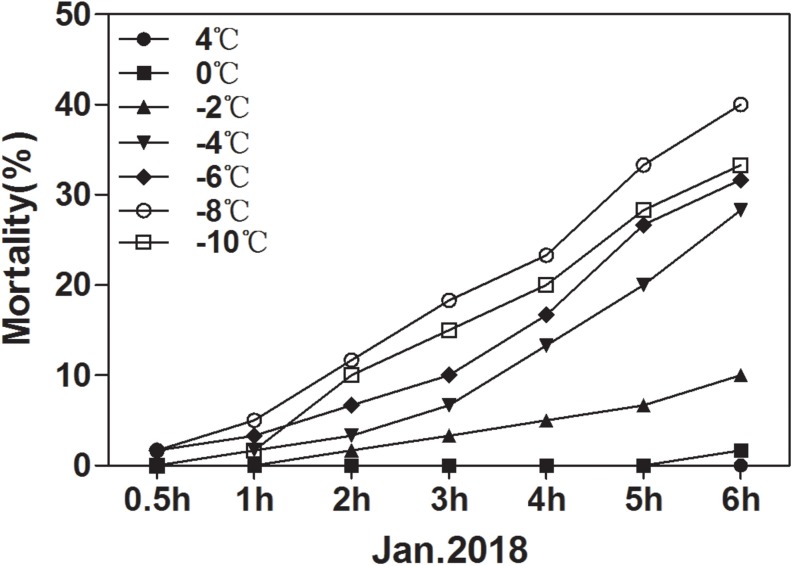
Mortality of *D. armandi* larvae at low temperatures. The larvae were treated at low temperatures (4, 0, –2, –4, –6, –8 and –10°C) for different time periods (0.5, 1, 2, 3, 4, 5, and 6 h) in January 2018.

The ability of overwintering *D. armandi* larvae to survive at low temperatures improved in January (Figure 7). In January, the larvae could tolerate temperatures of –10°C for 6 h, but mortality was 33.33%. It was noted that the lower the temperature at longer durations, the higher the observed mortality. However, mortality at –8°C was higher than –10°C after 6 h (Figure 7). Our results confirm a higher cold tolerance of larvae because they were able to survive when exposed to low temperatures, and when the temperature is low enough, the mortality rate drops.

### Efficiency Analysis of RNAi on DaAqps

#### Effect of dsRNA Treatment on DaAqps Transcript Level

**FIGURE 8:**
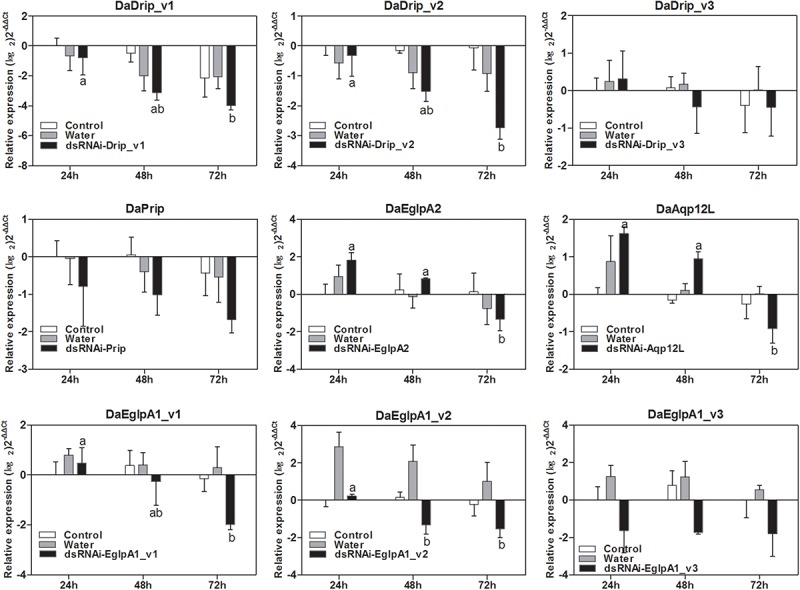
qRT-PCR analysis of DaAqps transcript patterns from *D. armandi* larvae after injected for 24, 48, and 72 h. Transcript patterns of DaAqps were analyzed on January 2018. The standard errors of the means of three biological replicates are represented by error bars. Significant differences between treatments are marked with letters (*p* < 0.05, one-way ANOVA). All values are mean ± *SE*, *n* = 3.

The relative expression level of DaAqps peaked in January 2018 (Figure 5). Therefore, we composed the dsRNA and injected it into the larvae in January to study the influence of RNAi on mortality. As shown in Figure 8, injection of dsRNA significantly decreased target gene expression. There were significant differences among the non-injected, water-injected and dsRNA-injected groups at 24, 48, and 72 h after dsRNA injection. DaAqps expression in the dsRNA-injected groups was down-regulated after 72 h.

#### Effect of dsRNA Treatment on Mortality Under Low Temperatures

**FIGURE 9:**
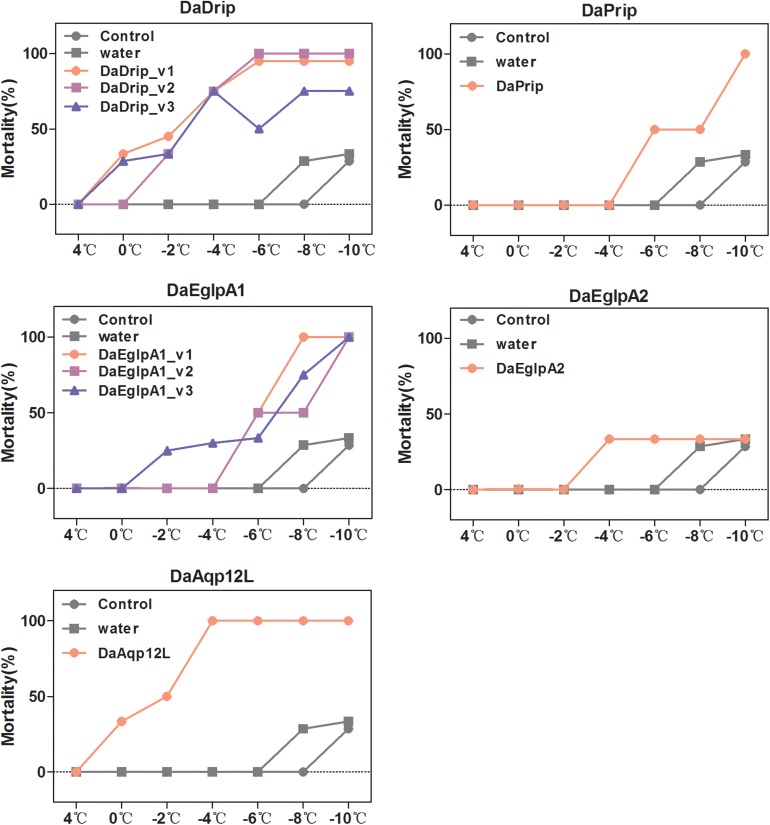
Mortality responses of RNAi *D. armandi* larvae to different low temperatures. Mortality responses of dsRNAi-treated, water-injected, and non-injected *D. armandi* larvae to different low temperatures (4, 0, –2, –4, –6, –8, and –10°C) for 1 h. Larvae were collected in January 2018. The mortality responses of non-injected and water-injected larvae were same in all DaAqps.

We tested the responses of dsRNA-injected, water-injected, and non-injected larvae to low temperature by mortality analysis. Mortality changed significantly with exposure to different low temperatures for different durations (Figure 7). We selected 1 h of exposure between 4 and –10°C to detect the effect of dsRNA treatment. After cold stress, the mortality of the dsRNA-treated larvae was higher than that of the water-injected and non-injected controls (Figure 9). An evident increase in mortality occurred when the larvae were injected with dsRNA from –6 to –10°C, except for DaEglpA2 (Figure 9).

## Discussion

Insect AQPs are essential for homeostasis because they regulate water movement. For example, AQPs are involved in desiccation and cold tolerance in gall flies, Diptera fergusoninidae (Philip et al., 2008, 2011), diuresis in mosquitoes (Drake et al., 2010; Liu et al., 2011), and heat tolerance and intrauterine lactation in Tsetse flies (Benoit et al., 2014). Insects have multiple functional AQPs and based on other systems these proteins probably play diverse physiological roles (Campbell et al., 2008; Gomes et al., 2009). However, comprehensive molecular and functional characterization of most insect AQPs is regarded as their initial identification. For example, *Drosophila melanogaster* has eight AQPs, but only two have been functionally described (Yool and Weinstein, 2002; Kaufmann et al., 2005). Furthermore, biochemical characterization of AQPs in mosquitoes is not yet complete, and the roles of these genes are yet to be phenotypically defined. To better understand the biological significance of AQP response to cold stress in bark beetles, we structurally and functionally identified some AQPs gene sequences.

Nine full-length genes were cloned from *D. armandi*. In total, five AQPs (DaDrip, DaPrip, DaEglpA1, DaEglpA2, and DaAqp12L) were determined with DaDrip and DaEglpA1 with three isoforms each. The sequence characteristics of DaAqps were similar to those of AQPs in other species. Two standard NPA motifs along with residues (the ar/R constriction site) were critical for traversing the AQP channel to determine the selectivity of solutes (Sui et al., 2001; Beitz et al., 2006). The residues form the narrowest restriction point and, similar to the AQP water channel, and it is only slightly larger than the size of a single water molecule (Sui et al., 2001; Beitz et al., 2006; Ho et al., 2009). It was found that residues participated in defining solute permeability and additionally influence the permeation pathways in the AQP channel (Wallace et al., 2012). It is possible that mature proteins may not have been exposed to glycosylation machinery because DaAqps lack a signal peptide. However, they have 6–7 transmembrane topology domains and many phosphorylation sites that also fit the pattern known for other members of MIP (Fabrick et al., 2014). In general, DaAqps sequence characteristics all conform to the typical AQP, which is found in different arthropod subfamilies (Campbell et al., 2008).

The phylogenetic alignments of DaAqps with other arthropod representatives were used to classify four subfamilies of arthropod AQPs, including two previously described (Drip and Prip) (Campbell et al., 2008), and two new subfamilies (Eglps and Aqp12L). Two functional classes of AQPs have previously been characterized within four phylogenetic subfamilies, including water-specific AQPs and Glps (Echevarría et al., 2001; Shakesby et al., 2009; Mathew et al., 2011). In DaAqps, the arthropod Drip subfamily contains a number of water-specific AQPs that generally improve fluid homeostasis by moving water through tissues to conserve osmotic potential (Campbell et al., 2008; Mathew et al., 2011). According to two conserved AQP motifs and sequence similarity (Figures 1, 2), two *O*-glycosylation and the predicted transmembrane domain (Table 3), and the three isoforms are water-specific AQPs in the Drip subfamily (Kaufmann et al., 2005). DaPrip belongs to the arthropod Prip subfamily with diverse physiological functions, such as water recycling for the cryptonephric rectal complex (Azuma et al., 2012), hydration and dehydration (Kikawada et al., 2008), and water homeostasis during oogenesis (Herraiz et al., 2011). DaEglpA1 and DaEglpA2 belong to the Eglp subfamily, which has a multifunctional transport channel, that of the Glps. They are important for water homeostasis in the formation of body fat (Kikawada et al., 2008), urine (Echevarría et al., 2001), water or urea excretion, homeostasis in the midgut and Malpighian tubules (Kataoka et al., 2009), and osmoregulation in aphids (Wallace et al., 2012). DaAqp12L is placed in the new subfamily Aqp12L. They share similarity with “S-aquaporins” deviating from the other AQP subfamilies (Nozaki et al., 2008; Wallace et al., 2012; Calvanese et al., 2013).

The levels of DaAqps transcripts differed through the developmental stages of *D. armandi* (Figure 3). Previous studies have also reported similar trends in other arthropods and found that the highest expression of CsDrip1 (*Chilo suppressalis*) was observed in the third instar larvae (Lu et al., 2018). RsAQP1 (*Rhipicephalus sanguineus*) mRNA transcripts have been observed in unfed larvae and adults in ticks (Balla et al., 2009), and louse AQPs were observed to be highly expressed in adults (Stavang et al., 2015). These findings support that AQPs play key roles in insect response to cold stress because it is the mature larvae that overwinter. In our study, DaAqp expression was found to be at its lowest during the egg stage (Figure 3), which disagrees with a previous study that detected high levels of LhAQP1, 3, and 4 transcripts in eggs of *Lygus hesperus* (Fabrick et al., 2014). However, in *R. sanguineus*, no RsAQP1 expression could be detected in the embryos. Therefore, these results indicate that water transport is not necessarily required in the egg stage (Balla et al., 2009). DaAqps were more abundant in adult females than males, which is in agreement with the results of a previous study regarding AgDrip1 of the mosquito *Anopheles gambiae* (Liu et al., 2011). We speculated that high expression in females might be connected with storing energy to invade host trees. Furthermore, DaAqps were also at higher levels in adults compared to those in pupae (Figure 3), and the results of the previous study on *C. suppressalis* suggested that CsDrip1 expression might be related to reproduction (Lu et al., 2018).

Levels of DaAqp transcripts varied in *D. armandi* larval tissues (Figure 4). Except for DaDrip_v3, AQPs were most present in the body fat, which is one of the most cold-tolerant tissues because glycerol accumulates in overwintering larvae (Izumi et al., 2006, 2007). The body fat actively participates in the exchange of metabolites and solutes with hemolymph (Wallace et al., 2012). However, low abundance was also observed in the fat body of several species (Liu et al., 2011; Philip et al., 2011). DaPrip and DaEglpA1_v1 were more highly expressed in the hindgut than other tissues of the digestive tract, which is consistent with the results observed for CsDrip185 (Figure 4). Interestingly, DaEglpA2, DaDrip_v3, DaEglpA1_v2, DaEglpA1_v3, and DaAqp12L were more highly expressed in the foregut, where the AQP gene has been expressed in other insects such as *Coptotermes formosanus* (Kambara et al., 2009). DaDrip_v1 and DaDrip_v2 transcripts were expressed in the midgut, suggesting a role in water absorbance from food (Kambara et al., 2009) (Figure 4). We speculate that DaAqps may play a role not only in water transport processes in the body fat but also in water recycling in the digestive tract.

The expression of DaAqps peaked in mid-winter (Figure 5), following an early winter (December), low point between October and January. We inferred that the low expression in early winter might be in preparation for the coming cold stress in mid-winter. In addition, there was a wider range of DaAqps expression in mid-winter 2017 than in early spring 2018, and the expression of all AQPs was down-regulated in late spring (Figure 5). This finding might provide a basis that AQPs play a crucial role during the overwintering period because they maintained water homeostasis at low temperatures. Among the different test times under cold conditions (Figure 6), trends in DaAqps expression were like a valley, in that expression levels for almost all DaAqps decreased at lower temperatures until a critical temperature threshold but then increased for a time before decreasing again. We also found that the expression levels of DaDrip_v3 and DaEglpA1_v2 were different from those of other AQPs, and their expression was not conspicuous under low temperatures. This indicates that they may not participate in water transport during winter. Based on all of our results, we suspected that other AQPs may be present in *D. armandi* and could be coordinated to tolerate low-temperature stress conditions. Some species exhibited similar trends under extreme temperatures such as *C. suppressalis*, in which the CsDrip1 transcript was significantly down-regulated under low-temperature stress (Lu et al., 2018).

According to the expression level of each month in the *D. armandi* larvae, we selected January to measure mortality. We observed higher cold tolerance in larvae based on their greater ability to survive when exposed to low temperatures (Figure 7). It was also verified in *Monochamus alternatus* (Ma et al., 2006) and *D. melanogaster* (Yi et al., 2007).

The importance of DaAqps for cold tolerance capacity of overwintering larvae was further confirmed by our RNAi experiment. RNAi of AQPs was successful in that it could be used as a functional genomic tool in *D. armandi* (Figure 8). This effect was also reported on the RNAi of the vitellogenin gene in adult honeybees *Apis mellifera* (Drake et al., 2010). We examined the mortality of water-injected, dsRNA-injected, and non-injected *D. armandi* larvae exposed to low temperatures for 1 h (Figure 9). The low-temperature mortality was significantly lower in the two controls than in the dsRNA-treated larvae. Therefore, silencing the target genes DaAqps not only suppressed their expression levels but also led to increased mortality due to cold stress, affecting the cold-tolerance capacity of larvae. This result agrees with those from previous studies on other species, such as *A. aegypti*, which has six putative AQPs and RNAi was used to show that three of these have apparent roles in the Malpighian tubules (Drake et al., 2010).

Water homeostasis is a critical function for insects, especially those suffering from extreme environmental conditions such as drought or cold stress. Water channel proteins are important for improving osmoregulation and reaching equilibrium of internal water and water lost to the environment. AQP has been previously studied in many plants and animals, but it is not well known in bark beetles. Therefore, in the present study we used molecular and physiological methods to clarify the identification of DaAqps and their important roles in cold tolerance. Although our results do not completely elucidate insect overwintering behaviors or the cold-tolerance strategies in *D. armandi*, they may act as a foundation for future studies and provide novel insights for developing new pest control methods.

## Data Availability

All datasets generated for this study are included in the manuscript and/or the Supplementary Files.

## Author Contributions

DF, LD, and HC designed the experiments and revised the manuscript. DF, HG, YS, and BL performed the experiments. DF analyzed the data and drafted the manuscript. All authors read and approved the manuscript for final submission.

## Conflict of Interest Statement

The authors declare that the research was conducted in the absence of any commercial or financial relationships that could be construed as a potential conflict of interest.

## Footnotes

1 https://www.ncbi.nlm.nih.gov/orffinder

2 http://web.expasy.org/protparam

3 http://www.cbs.dtu.dk/services/SignalP/

4 http://www.cbs.dtu.dk/services/

5 https://blast.ncbi.nlm.nih.gov/Blast.cgi

6 http://espript.ibcp.fr/ESPript/cgi-bin/ESPript.cgi

7 http://www.cbs.dtu.dk/services/TMHMM/

8 http://www.cbs.dtu.dk/services/NetPhos/
