# Supplementary material for: Identification, Expression Patterns and RNA Interference of Aquaporins in Dendroctonus armandi (Coleoptera: Scolytinae) Larvae During Overwintering
Source: Front Physiol. 2019 Aug 2;10:967. doi: 10.3389/fphys.2019.00967 (PMC6688586; doi:10.3389/fphys.2019.00967)
Supplement: Supplementary file 1 [file Table_1.DOCX]

Supplementary Material

**Table S1** Primer sequences used in the research.

| Gene | Primer direction  5′ → 3′ | | purpose |
| --- | --- | --- | --- |
|  | Forward | Reverse |  |
| DaPrip | GCCTGACGAACCTCAACCCG | CCAAAGCAACAGCCAAACCA | cDNA |
|  | GGCTTTGTCTTGGTGTTGGTTGT | CCATTGGTTTGGCTGTTGCTTT | 3’RACE |
|  | TGTCCCAAAGCAACAGCCAAACCA | GCAGGGGAGTGATTTTCGGGTTGAGG | 5’ RACE |
|  | GGGCTCATTCAAAGTTCCACCAT | TCTTCCTTGTCCTTTATTCCGT | Full-length |
|  | GGAGTATGCGACCCAAACAGG | ATCAAAGCAGCAACCACCC | qPCR |
|  | TAATACGACTCACTATAGGGACCTCAATCCAGCCGTAACAA | TAATACGACTCACTATAGGGTGGCCCTACCCAATAAACCCA | RNAi |
| DaDrip_v1 | TTTACTGCCCGCTTCGTT | GAGGTCGTTCCTTCTTCC | cDNA |
|  | GGCTTTACTGAAGTGGCTCCCGA | TTTGCGATGGAAGAAGGAACGACC | 3’RACE |
|  | GGTCGTTCCTTCTTCCATCGCAAA | GTAAAGCCCAAGTTGCCGTTGATGAA | 5’ RACE |
|  | GGGGTAGTTGCGTAAATCCCATCA | CTGATTTATTTACAAGCCTACGC | Full-length |
|  | GGGTAGTTGCGTAAATCCCATCA | GTGCCAATAAACTCAGCAGCCAG | qPCR |
|  | TAATACGACTCACTATAGGGGGCCTGAACGAGATCACAGAAA | TAATACGACTCACTATAGGGCATAACCACGGAAGGACCAAAA | RNAi |
| DaDrip_v2 | TTTACTGCCCGCTTCGTT | GAGGTCGTTCCTTCTTCC | cDNA |
|  | GGCTTTACTGAAGTGGCTCCCGA | TTTGCGATGGAAGAAGGAACGACC | 3’RACE |
|  | GGTCGTTCCTTCTTCCATCGCAAA | GTAAAGCCCAAGTTGCCGTTGATGAA | 5’ RACE |
|  | CGCCACTGAAAAAACCAAATC | ACAAGCCTACGCTTAAGGGTATG | Full-length |
|  | CCGAGACTGTGAGACACGACA | CGGATTTCCCAGGCAAGA | qPCR |
|  | TAATACGACTCACTATAGGGATGCTGGCTGCTGAGTTTAT | TAATACGACTCACTATAGGGATAACCACGGAAGGACCAAAAG | RNAi |
| DaDrip_v3 | TTTACTGCCCGCTTCGTT | GAGGTCGTTCCTTCTTCC | cDNA |
|  | GGCTTTACTGAAGTGGCTCCCGA | TTTGCGATGGAAGAAGGAACGACC | 3’RACE |
|  | GGTCGTTCCTTCTTCCATCGCAAA | GTAAAGCCCAAGTTGCCGTTGATGAA | 5’ RACE |
|  | AATAAGTCAAGTGCGTAAAGCC | ACAAGCCTACGCTTAAGGGTATGCG | Full-length |
|  | TTTGGTCCTTCCGTGGTTAT | TACGAGTCATTGTCGCCTTT | qPCR |
|  | TAATACGACTCACTATAGGGGCCTGAACGAGATCACAGAAAA | TAATACGACTCACTATAGGGATAACCACGGAAGGACCAAAAG | RNAi |
| DaEglpA1_v1 | ATGTTCCTCGGATGTATGG | AAAGTTACAGCAGGGTTGA | cDNA |
|  | GCTACACATCATTTGCCGTCACT | GCCCACCTCAACCCTGCTGTAACT | 3’RACE |
|  | TATCCCAAACGCCGCAACAAACCAA | AAAGTTACAGCAGGGTTGAGGTGGG | 5’ RACE |
|  | GTGCGCTTGTTTATTCTGTG | CGGTGTGGAATCCTGCTTATGCTCA | Full-length |
|  | GCGCTTGTTTATTCTGTGA | TATTTCCGTCTTGGCATC | qPCR |
|  | TAATACGACTCACTATAGGGAGGTCTCAAGAAGGAATGCCAG | TAATACGACTCACTATAGGGAACCGAATACAAACCGCAGCCG | RNAi |
| DaEglpA1_v2 | ATGTTCCTCGGATGTATGG | AAAGTTACAGCAGGGTTGA | cDNA |
|  | GCTACACATCATTTGCCGTCACT | GCCCACCTCAACCCTGCTGTAACT | 3’RACE |
|  | TATCCCAAACGCCGCAACAAACCAA | AAAGTTACAGCAGGGTTGAGGTGGG | 5’ RACE |
|  | GCTTTTAACCTCCGATTCAATATGT | AAATAGTTTTGCCACTTCGTAAC | Full-length |
|  | GACAAAGTAAATCGGCAAAACGCA | CCAAAAGAAAGTGACGGCAAATGA | qPCR |
|  | TAATACGACTCACTATAGGGCTAATGTTCCTCGGATGTATGG | TAATACGACTCACTATAGGGCCCAAACGCCGCAACAAACCAA | RNAi |
| DaEglpA1_v3 | ATGTTCCTCGGATGTATGG | AAAGTTACAGCAGGGTTGA | cDNA |
|  | GCTACACATCATTTGCCGTCACT | GCCCACCTCAACCCTGCTGTAACT | 3’RACE |
|  | TATCCCAAACGCCGCAACAAACCAA | AAAGTTACAGCAGGGTTGAGGTGGG | 5’ RACE |
|  | TGGAACTTGTGGTGTCGTGA | AAATAGTTTTGCCACTTCGTAAC | Full-length |
|  | TAATGGGATATGGAAAAGGTGA | AAGTTACAGCAGGGTTGAGGTG | qPCR |
|  | TAATACGACTCACTATAGGGCACATCATTTGCCGTCACTTTC | TAATACGACTCACTATAGGGTCCTGCTTATGCTCATTTCGTT | RNAi |
| DaEglpA2 | GGAACCCTTGTTAAATCTGC | CGAATCATGCTTGTCGCTAT | cDNA |
|  | GGTTTTCTGGTGGAGTTCTTGGC | GGGATCAGCGAAATAGCGACAAG | 3’RACE |
|  | TTAGCACCAGTGTAGGGTCCAGCAGC | TCCCAAACTCCACAGCATACCAAAGC | 5’ RACE |
|  | ATCGGTGTGTGTGGTTAGTAGAA | GCCCATTAGAACACAAGCAACTGA | Full-length |
|  | GCCGTTGTAGGCTTTGGACT | GCTTGTCGCTATTTCGCTGA | qPCR |
|  | TAATACGACTCACTATAGGGGGGATGTATGGGCTGTGTT | TAATACGACTCACTATAGGGCCTTCCAGTCTCCGTTGAA | RNAi |
| DaAqp12L | ACAGCCTGTCCCTATTGCC | TTTGTCCTTGTGGGTTTCC | cDNA |
|  | TGATGGCAGCCCTAACAACTT | TGGAAACCCACAAGGACAA | 3’RACE |
|  | CGTAGGCTTTGTCCTTGTGGGTTTCC | CATGGGGCAATAGGGACAGGCTG | 5’ RACE |
|  | GGGGGCTGCCATTCATTTTCTTT | TTATTCTTCTTTGCCTCGCTTCA | Full-length |
|  | TTGCCACCGTTATGGTCGTTT | ATTCTTCTTTGCCTCGCTTCA | qPCR |
|  | TAATACGACTCACTATAGGGAGGTAATAGCAAGAAGCACAACG | TAATACGACTCACTATAGGGAAAGCCAAAACGACCATAACGGT | RNAi |
